# Analysis of postural control through nonlinear methods in older adults: a randomized controlled trial

**DOI:** 10.7717/peerj.21706

**Published:** 2026-09-17

**Authors:** Carolina A. Cabo, Mário C. Espada, Jose A. Parraca, Sara Santos, Bruno Gonçalves, Orlando Fernandes

**Affiliations:** 1 Departamento de Desporto e Saúde, Escola de Saúde e Desenvolvimento Humano, Universidade de Évora, Évora, Portugal; 2 Comprehensive Health Research Centre (CHRC), Universidade de Évora, Évora, Portugal; 3 Escola Superior de Educação, Universidade Politécnica de Setúbal, Setúbal, Portugal; 4 Sport Physical Activity and Health Research & Innovation Center (SPRINT), Instituto Politécnico de Santarém, Rio Maior, Portugal; 5 Life Quality Research Centre (CIEQV), Universidade Politécnica de Setúbal, Setúbal, Portugal; 6 Centro Interdisciplinar para o Estudo da Performance Humana (CIPER), Faculdade de Motricidade Humana, Lisboa, Portugal; 7 Department of Sport Sciences, Faculty of Sport and Health Sciences, Fit Generation Research Institute, Andorra la Vella, Andorra; 8 Departamento de Fisioterapia, Escola Superior de Saúde, Universidade do Algarve, Faro, Portugal

**Keywords:** Older adults, Motor control, Postural stability, Fall prevention, Postural sway

## Abstract

**Background:**

Human balance and body orientation are regulated by the postural control system, which plays a critical role in stability, particularly with advancing age.

**Objective:**

This study examined the effects of a sensorimotor training program on postural control in older adults (55–80 years), investigating how targeted exercise may influence postural regulation.

**Methods:**

A total of 86 community-dwelling individuals aged 55 and 80 years participated in the study and were randomly assigned to a Control Group (CG, *n* = 43; average age: 73.50 ± 6.08 years) and an Experimental Group (EG, *n* = 43; average age: 72.40 ± 7.03 years). The intervention lasted six months, with sessions twice a week, and consisted of six exercise circuits focused on sensorimotor stimulation. Each circuit included eight physical exercises, progressively adjusted in difficulty over time, according to predefined performance-based criteria and under the supervision of a trained instructor to ensure appropriate individual adaptation.

**Results:**

Analysis of covariance (ANCOVA) analyses adjusted for baseline values revealed selective between-group differences. Approximate Entropy (ApEn) showed a significant effect in mediolateral sway under eyes-open conditions (ApEn_ML_OE: *F* = 8.7, *p* = 0.004, *η*^2^*p* = 0.10). Lyapunov Exponent (LyE) showed significant effects under eyes-closed conditions in both anteroposterior and mediolateral directions (LyE_AP_CE: *F* = 4.0, *p* = 0.049; LyE_ML_CE: *F* = 13.6, *p* = 0.001), indicating improved local dynamic stability. No significant between-group differences were observed for Detrended Fluctuation Analysis (DFA). Substantial inter-individual variability was observed across participants.

**Conclusions:**

Sensorimotor training was associated with selective changes in nonlinear postural control measures, particularly under reduced visual input. However, responses were heterogeneous, indicating that effects were not uniform across individuals. These findings suggest that targeted exercise interventions may modulate specific aspects of postural control in older adults, with potential relevance for balance regulation and fall prevention.

## Introduction

Human movement and postural control are governed by complex, nonlinear dynamics, in which the relationship between input and output is not proportional but adaptive and context dependent. Nonlinear systems, often discussed in the context of “nonlinear dynamics,” exhibit behaviors that are variable, flexible, and sometimes chaotic. This inherent variability is a hallmark of healthy motor function, reflecting the capacity of the neuromuscular system to adapt to changing environmental and task demands. In contrast, reduced variability is often associated with rigidity and impaired adaptability (Harbourne & Stergiou, 2009).

Nonlinear approaches provide important insights into postural control by capturing its complexity, adaptability, and dynamic structure, aspects that are particularly relevant to fall risk in older adults. Integrating these perspectives allows for a more comprehensive understanding of postural regulation, especially in aging populations. From this perspective, variability in motor control is not merely a source of “noise,” but an essential feature of an adaptable and resilient system (Harbourne & Stergiou, 2009; Stergiou & Decker, 2011).

Aging is associated with declines in sensory, motor, and cognitive systems that can compromise balance. These changes include reductions in muscle strength, joint flexibility, proprioceptive sensitivity, and visual and vestibular processing speed, all of which contribute to impaired postural control (Mancini & Horak, 2010; Rubenstein, 2006). Because postural control relies on the integration of multiple sensory inputs, deficits in sensory-motor processing can increase sway and fall risk.

To evaluate the contribution of sensory systems to postural control, balance was assessed under both eyes-open and eyes-closed conditions. Eyes-open trials reflect natural balance control with full visual feedback, whereas eyes-closed trials reduce visual input, increasing reliance on proprioception and vestibular signals. This design allows identification of potential sensory-specific adaptations induced by the intervention (Sarvari et al., 2024).

Inactivity is a major factor accelerating age-related decline in postural control. Sedentary behavior exacerbates muscle atrophy, reduces joint mobility, and impairs cardiovascular and neuromuscular function, increasing the risk of falls and functional decline (Paterson & Warburton, 2010). Numerous studies have shown that older adults who engage in little to no physical activity have an increased risk of developing frailty, which is characterized by weakness, unintentional weight loss, and fatigue (Clegg et al., 2013). Moreover, inactivity is closely linked to a higher incidence of falls, hospitalization, and a decline in overall quality of life. Although the present study did not specifically recruit sedentary individuals, the participants—community-dwelling and functionally independent older adults—represent a population in which decreased levels of physical activity are common. Engaging in regular exercise, particularly programs that emphasize strength, flexibility, and balance, can mitigate the effects of aging by preserving or even enhancing neuromuscular function and postural stability (Sherrington et al., 2019).

Nonlinear measures provide sensitive tools for detecting subtle changes in postural control that may not be apparent in linear metrics alone (Amirpourabasi et al., 2026; Lockhart & Liu, 2008; Rhea et al., 2019). Approximate entropy (ApEn) quantifies the regularity and predictability of sway patterns, with lower values indicating a more rigid system and higher values reflecting greater adaptability and resilience to perturbations (Rhea et al., 2011; Yentes et al., 2013). Thus, entropy serves as a valuable metric in understanding the dynamic nature of postural control, especially in populations at risk of falls, such as older adults.

Fractal dimension (FD) is another important measure that captures the complexity of a time series by examining how detail changes with scale. The concept of fractality, when applied to postural control, provides insights into the underlying structure and coordination of movement patterns. In this study, three algorithms were used to calculate FD: the Box-Counting Method, Higuchi’s Method, and Katz’s Method. These methods allow a nuanced evaluation of postural complexity, complementing entropy and traditional sway measures, and are particularly useful in assessing balance adaptations in older adults (Katz, 1988; Kirkby, 1983; Phinyomark, Larracy & Scheme, 2020).

In addition to entropy and fractal dimension, Detrended Fluctuation Analysis (DFA) of the Center of Pressure (CoP) provides valuable information about the intensity and dynamics of postural sway. Higher DFA values indicate rapid, potentially uncontrolled sway movements, reflecting reduced stability and increased fall risk, whereas lower values suggest smoother, more controlled postural adjustments (Tipton, Alderink & Rhodes, 2023). Including DFA alongside entropy and fractal dimension allows a more comprehensive assessment of both the temporal and spatial aspects of balance control, enhancing the understanding of postural stability in older adults (Amoud et al., 2007).

Despite extensive research on balance in older adults, gaps remain in understanding how structured sensorimotor training influences nonlinear postural control. While numerous studies have examined young adults or clinical populations, few have applied a combination of ApEn, FD, and DFA to track exercise-induced adaptations in community-dwelling older adults (Higgins et al., 2021; Mancini & Horak, 2010). In particular, the mechanisms by which sensorimotor exercises modify sway regularity, complexity, and stability remain poorly described.

Therefore, this randomized controlled trial (RCT) addresses these gaps by investigating the effects of a structured six-month sensorimotor training program on nonlinear indices of postural control in community-dwelling older adults. The intervention targeted the sensorimotor system directly, combining proprioceptive, vestibular, and balance-challenging exercises, and was designed according to FITT principles (Frequency, Intensity, Time, and Type) to allow progressive adaptation at the individual level (Sherrington et al., 2019). We hypothesized that the experimental group would show enhanced sway complexity, improved temporal organization, and greater adaptability in balance control compared with a control group, particularly under conditions of reduced sensory input (eyes closed). Specifically, complexity and temporal organization were assessed using Approximate Entropy (ApEn), local dynamic stability using the Lyapunov Exponent (LyE), and long-range temporal correlations using DFA. This combined analytical approach aims to provide insight into how targeted exercise interventions can modulate postural stability and reduce fall risk in older adults (Ness, Gurney & Ice, 2003). Such adaptations are expected to emerge through repeated sensory challenges, promoting improved integration of visual, vestibular, and proprioceptive inputs.

## Materials & Methods

### Data collection

The study was approved by the Ethics Committee of the University of É vora (approval number: 21040).

The study was conducted in accordance with the ethical principles outlined in the Declaration of Helsinki and followed the established ethical standards for research in sports sciences. Written informed consent was obtained from all participants prior to their inclusion in the study. The protocol was registered in the ClinicalTrials.gov PRS system (Registration Number: NCT05398354; https://www.clinicaltrials.gov/ct2/show/NCT05398354?term=NCT05398354&draw=2&rank=1).

### Participants

**Figure 1:**
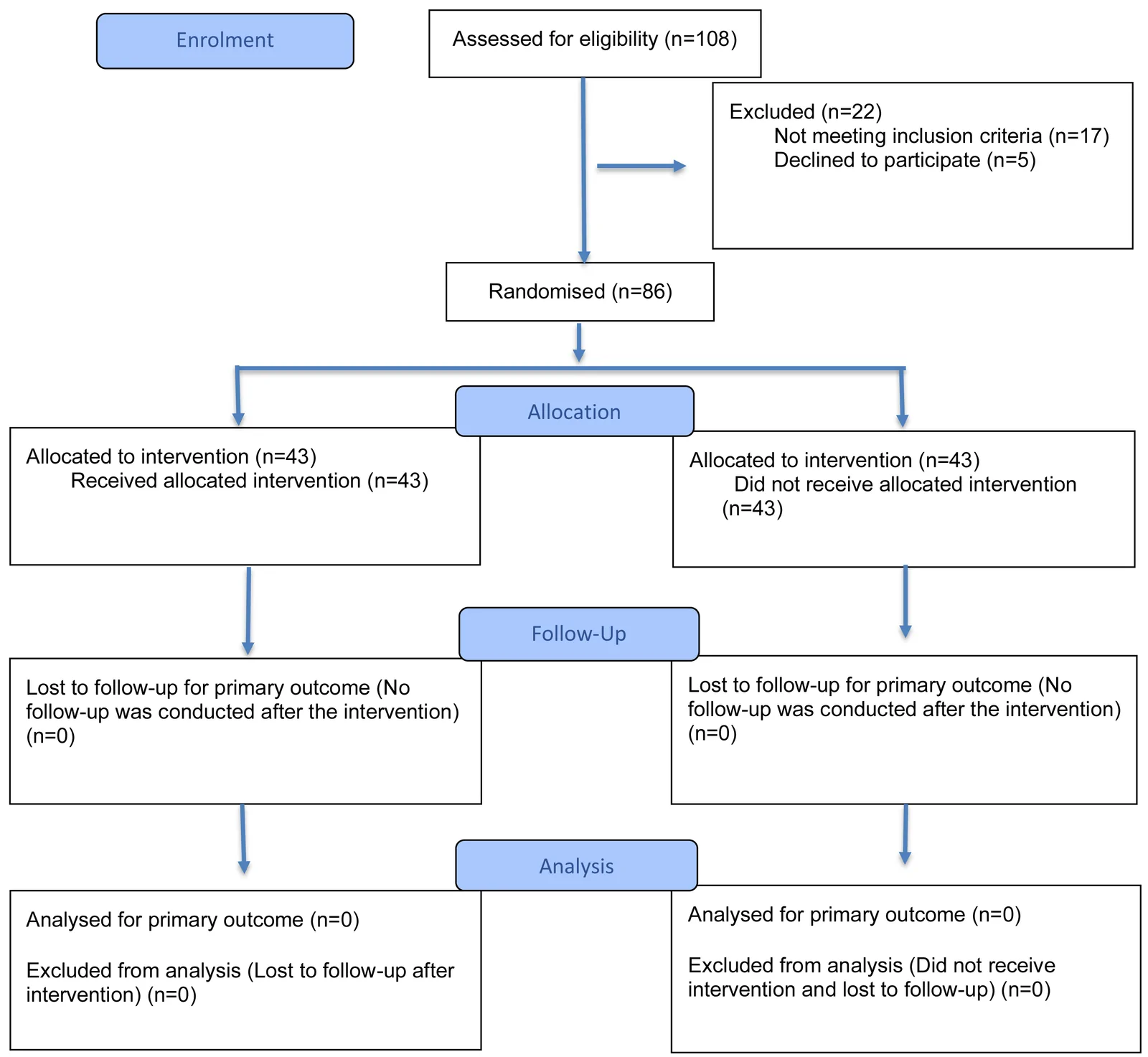
CONSORT flow diagram.

A total of 108 individuals were initially recruited for this randomized controlled trial, of which 86 met the eligibility criteria and were included in the final analysis (Fig. 1). Participants were independent, living in the municipality of Almada (Portugal), without institutionalization or specialized care. Twenty-two participants were excluded based on predefined criteria. Eligible participants were randomly assigned to the Experimental Group (EG, *n* = 43) or Control Group (CG, *n* = 43) using a computer-generated randomization sequence, with allocation concealed until the start of the intervention (Table 1). This procedure minimized allocation bias and ensured comparable baseline characteristics between groups.

Inclusion criteria required participants to be within the target age range, without recent surgical procedures (within the last six months), and not using prosthetic devices (excluding dental prostheses). Exclusion criteria included musculoskeletal conditions, impaired mobility (*e.g.*, use of a wheelchair or inability to walk independently for at least 15 min), psychiatric or neurological conditions, and clinically diagnosed cardiovascular disease.

Sample size estimation was performed using G*Power version 3.1.9.4 (Kiel University, Kiel, Germany), based on a two-tailed independent samples *t*-test. Assuming an alpha level of 0.05, statistical power of 0.80, and an effect size of Cohen’s *d* = 0.65 (based on previous literature), a total sample size of 86 participants (43 per group) was required. Although the primary analysis was conducted using ANCOVA adjusting for baseline values, the sample size was estimated using a *t*-test approach, which provides a conservative approximation.

### Experimental design

Data acquisition was conducted using a compact, wireless force platform (dimensions: 45  × 45 cm) featuring four load sensors and linked to a signal recording unit (PLUX^®^, Lisbon, Portugal), equipped with four load sensors and connected to a signal acquisition unit. Signals were recorded at 1,000 Hz and subsequently down sampled by a factor of 20, resulting in an effective sampling frequency of 50 Hz for analysis. The center of pressure (CoP) signals were filtered using a fourth-order zero-lag low-pass Butterworth filter with a 10 Hz cutoff frequency, in accordance with established procedures (Hernandez et al., 2016). Participants stood barefoot with feet shoulder-width apart and arms relaxed, maintaining a consistent posture across trials. Three trials of 120 s each were conducted under:

**Table 1:** Characteristics of study participants.

** **	**Exercise group**	**Control group**
Total	43	43
Men	13	10
Women	30	33
Age	72.40 (± 7.03)	73.50 (± 6.08)
BMI	27.00 (± 5.27)	27.90 (± 4.45)

• Open eyes (OE): gaze fixed on a visual marker at eye level, allowing full visual input.
• Closed eyes (CE): eyes fully closed, isolating proprioceptive and vestibular contributions.

CoP trajectories were centered relative to their mean (Prieto et al., 1996; Qiu & Xiong, 2015), and consistent foot positioning was ensured across trials to minimize variability.

In addition to nonlinear metrics, linear postural control variables (*e.g.*, sway amplitude and displacement measures) were calculated from the CoP trajectories to characterize the magnitude of postural sway.

### Nonlinear variables

The parameters employed were embedding dimension (m) = 2, tolerance (r) = 0.25, and time delay (tau) = 6, which was determined *via* the Average Mutual Information (AMI) method. The number of data points analyzed exceeded 2,000 (*N* > 2,000). The tolerance parameter (r) was defined as 0.25 times the standard deviation of the time series, in accordance with commonly recommended values (0.1–0.25 × SD) for physiological signals, ensuring robustness of entropy estimates in the presence of noise. Although Approximate entropy can be computed without a time delay, a delay (*τ*) was incorporated to reduce redundancy between consecutive data points and improve phase-space reconstruction. The time delay (*τ* = 6) was determined using the first minimum of the Average Mutual Information (AMI) function, a standard approach in nonlinear time-series analysis (Govindan et al., 2007; Kaffashi et al., 2008).

Approximate Entropy (ApEn):

Approximate entropy was calculated to quantify the regularity and temporal organization of CoP fluctuations. Although ApEn is more sensitive to data length and noise, it still serves as a valuable marker of postural adaptability.

• ApEn_AP: Analyzed in the anteroposterior direction under both open eyes (ApEn_AP_OE) and closed eyes (ApEn_AP_CE) conditions.
• ApEn_ML: Analyzed in the mediolateral direction under both open eyes (ApEn_ML_OE) and closed eyes (ApEn_ML_CE) conditions.

Lyapunov Exponent (LyE):

The Lyapunov exponent was used to assess local dynamic stability, reflecting how the system responds to small perturbations over time. Higher values typically indicate reduced stability.

• LyE_AP: Analyzed in the anteroposterior direction under both open eyes (LyeEn_AP_OE) and closed eyes (LyeEn_AP_CE) conditions.
• Lye_ML: Analyzed in the mediolateral direction under both open eyes (LyeEn_AP_OE) and closed eyes (LyeEn_AP_CE) conditions.

Detrend Fluctuation Analysis (DFA):

DFA is a nonlinear statistical method used to detect long-range temporal correlations in non-stationary time series—*i.e.,* signals whose mean or variance changes over time.

Real-world data (*e.g.*, heart rate, stock prices, climate data) are often non-stationary. The linear datasets considered here were previously employed in a related study (Cabo et al., 2026), and are reused in the present work to facilitate comparison with established linear approaches.

DFA avoids spurious results by eliminating local trends before assessing correlation.

• aAP: Analyzed in the anteroposterior direction under both open eyes (aAP_OE) and closed eyes (aAP_CE) conditions.
• aML: Analyzed in the mediolateral direction under both open eyes (aAP_OE) and closed eyes (aAP_CE) conditions.

Nonlinear measures were calculated from the Center of Pressure (CoP) time series to assess postural control dynamics beyond simple linear parameters. These nonlinear metrics were computed separately for anteroposterior (AP) and mediolateral (ML) directions, under both eyes-open (OE) and eyes-closed (CE) conditions, following the procedures described by Quijoux et al. (2021) (Table 2).

**Table 2:** Mathematical definitions, algorithmic descriptions, and parameter values of nonlinear measures.

**Metric**	**Measures**	**Key Equation/Formula**
**Approximate Entropy (ApEn)**	Signal regularity and predictability (with bias)	ApEn(m, r) = Φ^*m*^(r)−Φ^*m*+^^1^ (r)
**Lyapunov Exponent (LyE)**	Sensitivity to initial conditions/local dynamic stability	*λ*= lim_*t*_→∞ (1/t) ln(*δ*(t)/*δ*_0_)
**Detrended Fluctuation Analysis (DFA)**	Long-range temporal correlations	F(n) ∼n^α^

### Experimental protocol

The sensorimotor intervention spanned 24 weeks, with two 45-minute sessions per week following FITT principles: Frequency = 2 sessions; Intensity = moderate (Borg CR10: 5–6); Time = 45 min; Type = multimodal exercises targeting balance, proprioception, coordination, and lower-limb strength.

**Figure 2:**
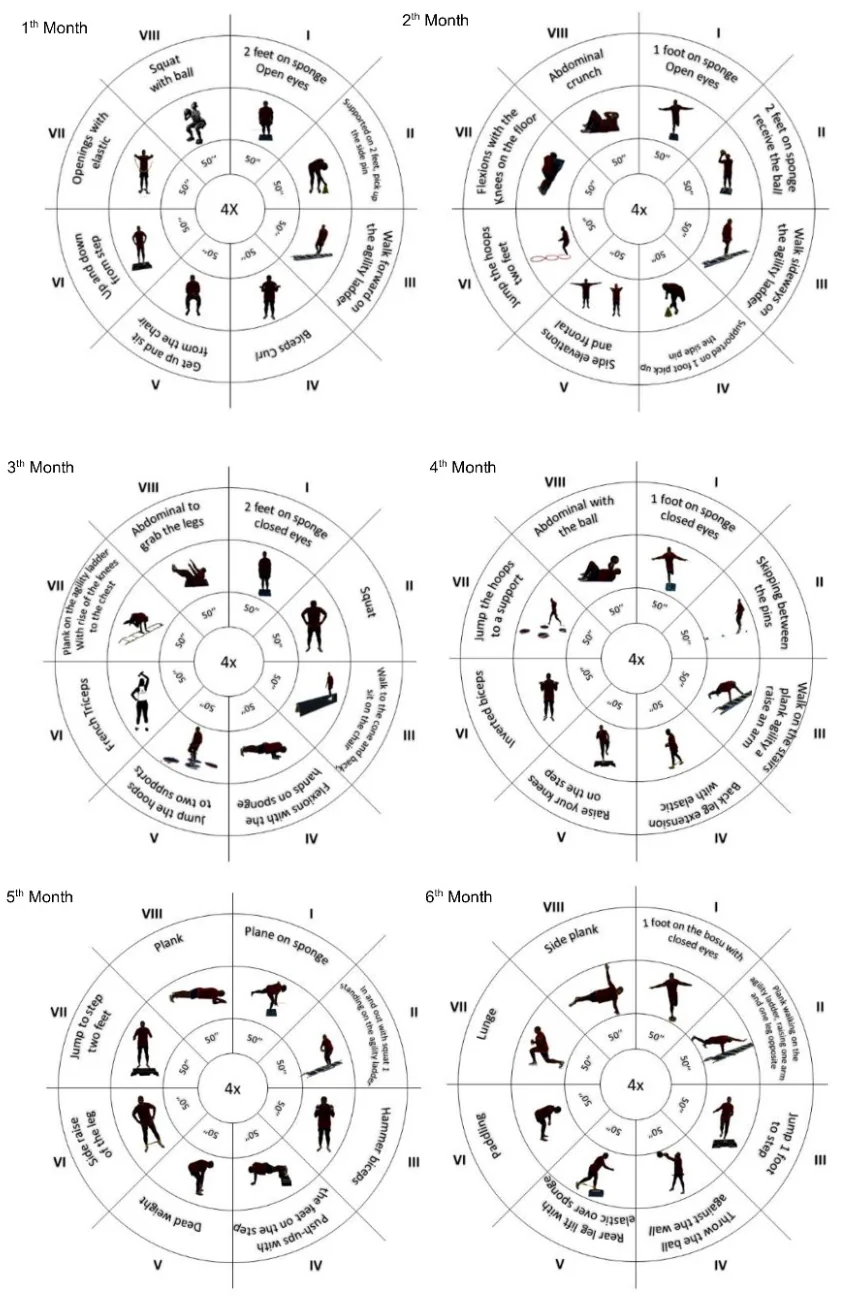
Main part of the sessions according to the month of the study (Cabo et al., 2022).

The training protocol followed a progressive model, beginning with bodyweight-only exercises during the initial eight weeks. From weeks 9 to 16, resistance tools such as elastic bands, ankle weights, and free weights were incorporated to increase challenge. In the final phase (weeks 17 to 24), the resistance was further intensified to promote continued adaptation (Fig. 2).

Each month introduced a variation in training focus, while every 45-minute session was structured into three phases: a 10-minute warm-up phase that began with a 5-minute walk along a flat, obstacle-free indoor path, followed by joint mobility exercises; a 25-minute core component consisting of four rounds of eight exercises performed in a circuit format (50 s of effort followed by 15 s of rest); and a 10-minute cooldown with muscle stretching routines. Exercise intensity and complexity were adjusted weekly, ensuring progression while maintaining adherence to FITT principles and individual safety guidelines.

Participants assigned to the control group maintained their usual daily activities and did not receive any structured exercise intervention during the study period. However, they were offered the opportunity to complete the same training protocol after the conclusion of the study. All assessments were carried out before the intervention began and repeated immediately following its completion. To ensure consistency and ease during data collection, a familiarization session was held prior to baseline testing, allowing participants to become comfortable with the procedures and equipment used.

### Data analysis

Data preprocessing was conducted using the EEGLAB toolbox within the MATLAB environment (MathWorks Inc., Natick, MA, USA). For the analysis of nonlinear dynamics, we selected approximate entropy due to its robustness and lower sensitivity to parameter variation, as described by Yentes et al. (2013).

Descriptive analyses for both the intervention and control groups were performed using Jamovi (Version 2.5.2.0). To assess data distribution, the Shapiro–Wilk test was applied, where *p*-values greater than 0.05 indicated a normal distribution, and *p*-values ≤ 0.05 suggested non-normality.

To examine within-group changes, paired-sample t-tests were applied. Intergroup analysis was performed using Analysis of Covariance (ANCOVA), with post-intervention scores as the dependent variable and baseline scores as covariates, to evaluate whether the intervention led to statistically significant differences between the Experimental Group (EG) and the Control Group (CG). *Post-hoc* pairwise comparisons were performed with Bonferroni correction to control Type I error. Effect sizes from ANCOVA were expressed as partial eta-squared (*η*^2^p), classified as small (0.01–0.059), medium (0.06–0.14), or large (>0.14). For *post-hoc* analysis and effect size interpretation, Cohen’s d was calculated along with 95% confidence intervals, categorized as small (0.2–0.49), medium (0.5–0.8), or large (>0.8), according to Calin-Jageman & Cumming (2017), Cohen (1988).

### Data availability

The datasets generated and analyzed in the present study are provided as supplementary material (raw data). The shared data corresponds to the processed datasets derived from the force platform recordings, including the calculated variables used in the statistical analyses. These datasets allow full verification of the analytical procedures and statistical results reported in the manuscript, while ensuring participant confidentiality. Raw force platform signal files are not included, as they contain identifiable metadata and require specific proprietary software for access and processing.

## Results

Postural control outcomes were analyzed using both within-group and between-group approaches. Between-group differences were assessed using ANCOVA adjusted for baseline values, while within-group changes were analyzed using paired-sample t-tests (Table 3).

**Table 3:** Paired sample T-Test.

**Paired Samples T-Test-Student’s t**		**Statistic (DF = 86)**	***p***		
ApEn_AP_OE_pre	ApEn_AP_OE_post	−2,649	0.01	*	
LyE_AP_OE_pre	LyE_AP_OE_post	−6,369	<0.001	***	
LyE_ML_OE_pre	LyE_ML_OE_post	−3,578	<0.001	***	
aAP_OE_pre	aAP_OE_post	4,483	<0.001	***	
LyE_AP_CE_pre	LyE_AP_CE_post	−5,067	<0.001	***	
LyE_ML_CE_pre	LyE_ML_CE_post	−2,391	0.019	*	
aAP_CE_pre	aAP_CE_post	4,556	<0.001	***	
aML_CE_pre	aML_CE_post	3,845	<0.001	***	

**Notes.**

H_*a*_ μ_Measure1−Measure2_ ≠ 0.

* *p* < 0.05.

*** *p* < 0.001

Data are presented as mean ± standard deviation (SD). Twenty-two participants were excluded based on predefined eligibility criteria, including age, recent surgery, use of prosthetic devices, musculoskeletal, neurological, or psychiatric conditions, impaired mobility, or clinically diagnosed cardiovascular disease. No technical issues or data loss occurred during data collection; excluded data were removed prior to analysis.

### Within-group comparisons

Within-group analyses were performed separately for the Experimental Group (EG) and Control Group (CG) using paired-sample t-tests (Table 3).

In the EG, significant pre–post differences were observed for several nonlinear parameters. Approximate Entropy (ApEn) showed significant changes in both visual conditions (ApEn_OE: *p* = 0.01; ApEn_CE: *p* = 0.005). Significant differences were also observed for Lyapunov Exponent (LyE) across all conditions (LyE_AP_OE: *p* < 0.001; LyE_AP_CE: *p* < 0.001; LyE_ML_OE: *p* < 0.001; LyE_ML_CE: *p* = 0.019). Detrended Fluctuation Analysis (DFA) showed significant changes in selected conditions (aAP_OE: *p* < 0.001; aAP_CE: *p* < 0.001; aML_CE: *p* < 0.001).

In the CG, fewer and less consistent within-group changes were observed across the same variables and conditions (Table 3).

### Between-group comparisons

Between-group differences were assessed using ANCOVA adjusted for baseline values (Table 4).

**Table 4:** Results of ANCOVA comparing postural control parameters between-group comparisons (adjusted pos*t*-test means with 95% CIs), supported by ANCOVA outputs (*p*-values, F-values, and partial *η*^2^ for each variable).

Variables	Control group				Experimental group				ANCOVA		
	Pre-test	Post-teste	Mean differences [95% CI]	Cohen’s d [95% CI]	Pre-test	Post-teste	Mean differences [95% CI]	Cohen’s d [95% CI]	F	*p*	Partial eta2
ApEn_AP_OE	0.68 ± 0.06	0.7 ± 0.06	0.02 [0.04; 0]	0.27 [0.58; −0.03]	0.68 ± 0.06	0.69 ± 0.05	0.02 [0.04; 0]	0.3 [0.6; −0.01]	0.1	0.716	0
ApEn_ML_OE	0.69 ± 0.09	0.67 ± 0.07	−0.03 [3.24; −0.05]	−0.3 [0; −0.61]	0.7 ± 0.08	0.72 ± 0.1	0.02 [0.04; −0.01]	0.23 [0.53; −0.07]	8.7	0.004^*^	0.1
LyE_AP_OE	14.82 ± 3.75	17.69 ± 3.45	2.87 [4.12; 1.62]^*^	0.71 [1.04; 0.37]	15.66 ± 4.51	18.41 ± 3.08	2.75 [4.03; 1.46]^*^	0.66 [0.99; 0.33]	0.5	0.494	0.01
LyE_ML_OE	13.32 ± 4.14	15.47 ± 4.24	2.15 [3.69; 0.61]^*^	0.43 [0.74; 0.11]	13.6 ± 5.65	15.32 ± 4.57	1.72 [3.28; 0.16]^*^	0.34 [0.65; 0.03]	0.1	0.77	0
aAP_OE	1.05 ± 0.17	0.97 ± 0.16	−0.09 [−0.03; −0.14]^*^	−0.49 [−0.17; −0.81]	1.04 ± 0.14	0.96 ± 0.13	−0.07 [−0.03; −0.12]^*^	−0.47 [−0.15; −0.78]	0	0.971	0
aML_OE	0.96 ± 0.16	0.93 ± 0.16	−0.04 [0.01; −0.09]	−0.26 [0.05; −0.56]	0.97 ± 0.18	0.95 ± 0.16	−0.02 [0.03; −0.08]	−0.14 [0.16; −0.44]	0.4	0.505	0.01
ApEn_AP_CE	0.67 ± 0.05	0.69 ± 0.05	0.02 [0.05; 0]^*^	0.36 [0.67; 0.05]	0.67 ± 0.07	0.69 ± 0.05	0.02 [0.04; 0]	0.26 [0.57; −0.04]	0.1	0.727	0
ApEn_ML_CE	0.69 ± 0.09	0.69 ± 0.09	0 [0.03; −0.03]	0.02 [0.32; −0.28]	0.65 ± 0.10	0.67 ± 0.11	0.02 [0.05; 0]	0.29 [0.59; −0.02]	0.1	0.742	0
LyE_AP_CE	15.12 ± 4.27	18.01 ± 3.48	2.89 [4.14; 1.63]^*^	0.71 [1.04; 0.37]	14.92 ± 3.45	16.37 ± 4.67	1.45 [2.61; 0.29]^*^	0.39 [0.69; 0.07]	4	0.049^*^	0.05
LyE_ML_CE	13.07 ± 4.41	15.89 ± 3.59	2.82 [4.2; 1.44]^*^	0.63 [0.95; 0.3]	13.13 ± 4.08	12.83 ± 4.69	−0.3 [1.18; −1.78]	−0.06 [0.24; −0.36]	13.6	0.001^*^	0.14
aAP_CE	1.05 ± 0.17	0.94 ± 0.16	−0.11 [−0.05; −0.17]^*^	−0.55 [−0.22; −0.87]	1.06 ± 0.16	0.99 ± 0.17	−0.07 [−0.02; −0.12]^*^	−0.43 [−0.11; −0.74]	1.9	0.177	0.02
aML_CE	1.02 ± 0.14	0.94 ± 0.16	−0.07 [−0.02; −0.13]^*^	−0.4 [−0.09; −0.71]	1.06 ± 0.17	0.98 ± 0.2	−0.07 [−0.02; −0.13]^*^	−0.43 [−0.11; −0.74]	0.3	0.617	0

**Notes.**

* *p* < 0.05.

Significant differences were observed for ApEn_ML_OE (*F* = 8.7, *p* = 0.004, *η*^2^p = 0.10), indicating group differences in mediolateral entropy under eyes-open conditions.

For LyE, significant between-group differences were found in eyes-closed conditions for LyE_AP_CE (*F* = 4.0, *p* = 0.049, *η*^2^p = 0.05) and LyE_ML_CE (*F* = 13.6, *p* = 0.001, *η*^2^p = 0.14).

No significant between-group differences were observed for DFA variables (all *p* > 0.17) or for LyE under eyes-open conditions (all *p* > 0.49).

### Effect size analysis

**Figure 3:**
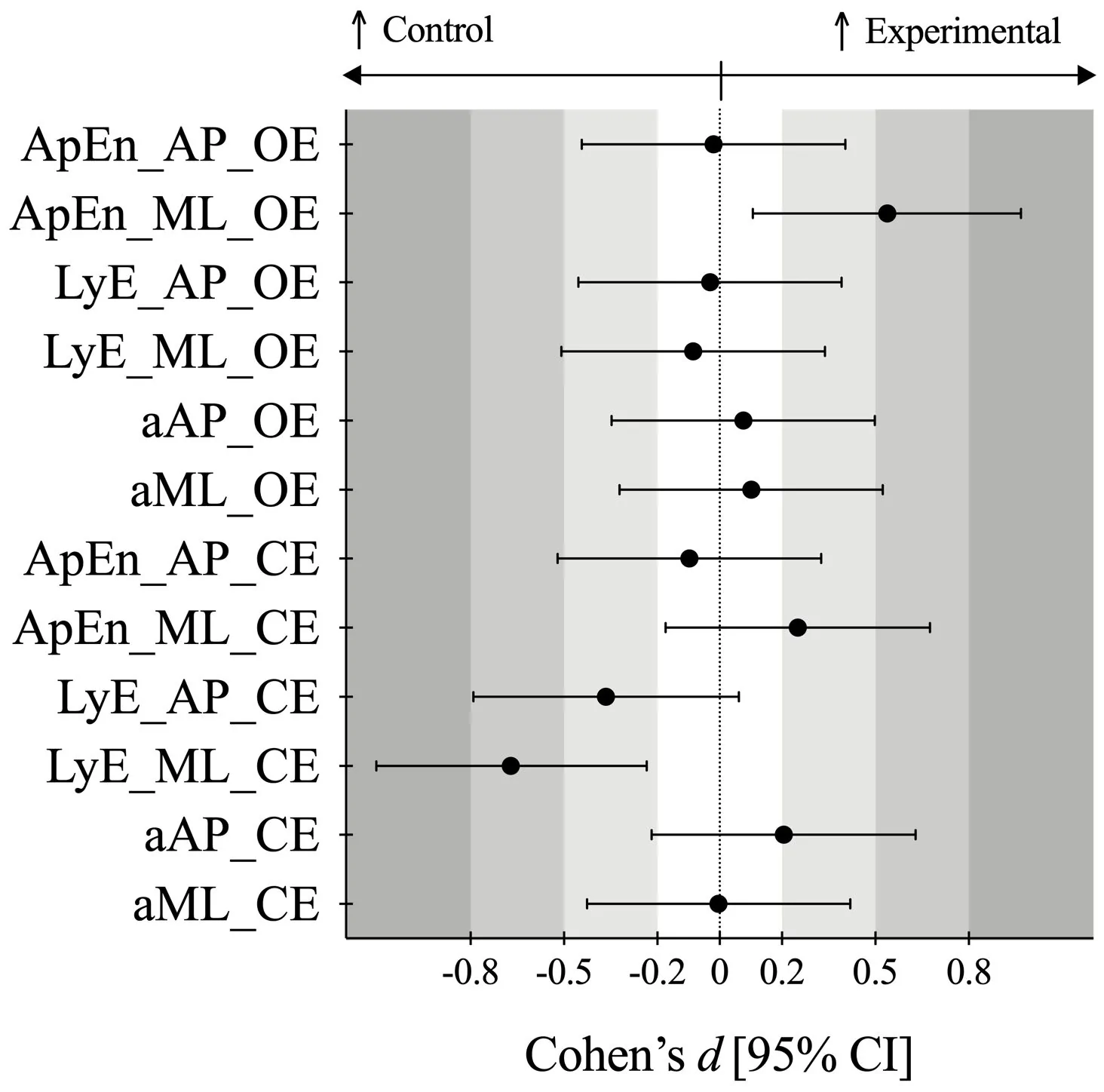
Cohen’s d differences between the Control and Experimental groups for the study variables. Error bars indicate uncertainty in the true mean changes, with 95% confidence intervals.

Standardized between-group effect sizes (Cohen’s d) are presented in Fig. 3. The largest effects were observed for LyE_ML_CE and ApEn_ML_OE, while most remaining variables showed small or negligible effects. Confidence intervals indicated variability in effect estimates across participants.

Overall, ANCOVA results indicated a limited number of statistically significant between-group differences in nonlinear postural control variables, primarily involving ApEn in the mediolateral direction and LyE under eyes-closed conditions. Within-group analyses showed more frequent changes in the experimental group compared with the control group; however, these findings were not confirmed across all variables in the between-group comparisons.

## Discussion

The purpose of this study was to examine the effects of a structured sensorimotor training program on postural control in older adults. Differences between the EG, who completed the intervention, and the CG were evaluated through CoP parameters in both the AP and ML directions, using nonlinear measures. Although this analytical framework provides a comprehensive view of postural stability, the generalizability of the findings remains limited by the sample size and participant characteristics.

We hypothesized that the sensorimotor intervention would lead to measurable improvements in the nonlinear characteristics of postural control, including increased complexity and regularity of sway patterns, enhanced local dynamic stability, and reduced postural DFA, reflecting better adaptability and stability in older adults. The findings partially supported this hypothesis and are consistent with previous studies showing improved balance control in physically trained older adults compared with sedentary individuals (Bacha et al., 2016; Costa et al., 2012; Rezende et al., 2012).

The observed changes in nonlinear measures suggest that sensorimotor training may induce selective adaptations in postural control rather than uniform improvements across all parameters. This pattern is consistent with previous evidence indicating that postural adaptations are task- and context-dependent, particularly in aging populations.

Previous studies have primarily compared physically active and inactive individuals; however, the present study extends this literature by applying a standardized nonlinear analytical framework, including consistent preprocessing procedures (CoP centering and 10 Hz low-pass filtering) and uniform outcome metrics (Approximate entropy, Lyapunov Exponent, and DFA). This methodological consistency enhances comparability across variables and with previous research (Figueiredo, Pereira & Neto, 2018; Shokouhyan et al., 2020). Nevertheless, the absence of functional performance outcomes limits direct conclusions regarding real-world functional implications.

Reductions in ApEn observed in the experimental group across visual conditions suggest increased regularity in postural sway. Lower entropy values have been interpreted as reflecting changes in neuromuscular control strategies (Roerdink, Hlavackova & Vuillerme, 2011) and are consistent with prior findings on the effects of sensorimotor training in older adults (Costa et al., 2012; Rezende et al., 2012). It is important to note that signal preprocessing, including low-pass filtering, may influence entropy-based measures by reducing high-frequency fluctuations (Lubetzky, Harel & Lubetzky, 2018), which should be considered when interpreting these findings.

Changes in DFA observed under reduced visual conditions may indicate alterations in temporal structure and sway regulation, consistent with previous reports linking lower DFA values to improved postural stability (Chiari, Rocchi & Cappello, 2002; Prieto et al., 1996). However, the absence of consistent between-group differences across all DFA measures suggests that these effects were not generalized across conditions.

Alterations in Lyapunov Exponent and entropy measures suggest modifications in the dynamic structure of postural control. These findings align with the notion that sensorimotor training may enhance the organization of postural responses, particularly under conditions of reduced visual input. AP-related measures appeared to show greater sensitivity to intervention-related changes, consistent with the recognized importance of anteroposterior control in functional balance tasks (Maki, Holliday & Topper, 1994; Peterka et al., 2018).

The nonlinear literature in aging populations shows heterogeneous findings, with reports of both increased and decreased entropy, Lyapunov, and fractal measures (Cavanaugh, Mercer & Stergiou, 2007; Manor et al., 2010; Duarte & Sternad, 2008; Kędziorek & Błazkiewicz, 2020). These inconsistencies highlight the context-dependent nature of nonlinear metrics and caution against linear interpretations of complexity-related changes. Within this framework, the present findings likely reflect adaptive reorganization of postural control rather than uniform improvement or deterioration.

From a neurophysiological perspective, sensorimotor training is grounded in neuroplasticity, whereby targeted sensory input promotes more efficient motor strategies (Kumar et al., 2023). The observed changes under eyes-closed conditions are consistent with increased reliance on proprioceptive and vestibular inputs when visual information is unavailable, potentially enhancing compensatory postural mechanisms (Matur & Oge, 2017). Improved stability under such conditions has been associated with reduced susceptibility to perturbations and fall risk (Mehdizadeh, 2018).

Substantial inter-individual variability was observed across participants, with divergent response patterns relative to group-level trends. This variability may reflect differences in baseline postural stability, motor strategies, or other unmeasured factors influencing responsiveness to training (Kędziorek & Błazkiewicz, 2020). These findings are consistent with the non-ergodic nature of postural control, where group averages may not fully represent individual adaptations. This highlights the importance of complementing group-level analyses with individual-level approaches in future research.

Overall, these results indicate that the intervention affected specific aspects of postural control dynamics rather than producing uniform changes across all nonlinear measures. From a theoretical standpoint, postural control can be understood as a complex, self-organizing system operating across multiple timescales. Nonlinear metrics such as entropy and Lyapunov exponents provide insight into the flexibility and adaptability of this system (Riley & Turvey, 2002; Stergiou & Decker, 2011). Changes in these measures should therefore be interpreted as shifts in system dynamics rather than strictly positive or negative adaptations.

From a practical perspective, the intervention frequency used in this study (twice weekly) aligns with international recommendations for physical activity in older adults (World Health Organization, 2020). Training under sensory-challenging conditions, such as eyes-closed tasks, may further enhance sensory reweighting mechanisms and improve balance control in populations at risk of instability.

From a clinical perspective, structured sensorimotor training may support improvements in postural control in older adults. However, these findings should be interpreted with caution due to sample size limitations, restricted participant characteristics, and the absence of long-term follow-up. In addition, the COVID-19 pandemic introduced logistical constraints, including safety procedures, spatial limitations, and transportation barriers, which may have influenced study implementation. The absence of data on participants’ fall history further limits clinical interpretation.

The absence of significant between-group differences for some variables may be related to limited statistical power associated with the sample size. Although an a priori power analysis was conducted, the observed variability in nonlinear outcomes suggests that the study may have been underpowered to detect small to moderate effects. Consequently, non-significant findings should be interpreted cautiously, as they may reflect type II error rather than absence of true intervention effects.

Future research should include larger and more diverse samples, longitudinal designs, and functional outcome measures to better establish the clinical relevance of sensorimotor training. In addition, the investigation of individual response profiles, including responder analysis and minimal detectable change, would further improve understanding of intervention effects.

## Conclusions

This study suggests that a structured, progressive sensorimotor training program may induce selective improvements in postural control in older adults, particularly under challenging sensory conditions such as eyes-closed tasks. Significant between-group differences were observed in mediolateral sway organization (ApEn_ML_OE) and local dynamic stability (Lyapunov Exponent under eyes-closed conditions), indicating intervention-related changes in postural control dynamics. These findings suggest that the sensorimotor training influenced the temporal structure and stability of postural sway in a context-dependent manner.

However, responses were heterogeneous across participants, suggesting inter-individual variability in adaptation to the intervention. This variability may be influenced by baseline postural control characteristics and individual responsiveness to training. Nonlinear metrics proved essential for detecting subtle changes that conventional linear measures might overlook, emphasizing their value in comprehensive balance assessment and intervention evaluation.

Overall, these findings highlight the value of nonlinear approaches and considering inter-individual variability when interpreting the effects of sensorimotor interventions on postural control in older adults.

##  Supplemental Information

10.7717/peerj.21706/supp-1 Supplemental Information 1 CONSORT checklist

10.7717/peerj.21706/supp-2 Supplemental Information 2 Raw data

## Additional Information and Declarations

### Data Availability

The following information was supplied regarding data availability: The data is available in the Supplemental Files.

### Clinical Trial Registration

The following information was supplied regarding Clinical Trial registration: NCT05398354
